# A Comparative Study between Air Bubble Tamponade Alone versus Air Bubble Tamponade with Internal Fluid Aspiration for Nonplanar Descemet's Membrane Detachment after Phacoemulsification

**DOI:** 10.1155/2021/9953418

**Published:** 2021-07-06

**Authors:** Amr A. Gab-Alla

**Affiliations:** Ophthalmology Department, Suez Canal University, Faculty of Medicine, Ismailia, Egypt

## Abstract

**Purpose:**

To compare the efficacy of air bubble tamponade alone versus air bubble tamponade with internal fluid aspiration for nonplanar Descemet's membrane detachment after clear corneal incision phacoemulsification.

**Methods:**

This study is a prospective, intervention, comparative randomised clinical trial, conducted at a private eye centre, Ismailia, Egypt, from March 2019 to March 2020. It contained 30 eyes of 24 patients who had postphacoemulsification nonplanar Descemet's membrane detachment involving the periphery and the central area of the cornea (>50% of the cornea) with corneal oedema. The patients were divided into two groups: group A: patients with nonplanar DMD affecting the central cornea treated by air bubble tamponade only and group B: patients with nonplanar DMD affecting the central cornea treated by air bubble tamponade augmented by internal fluid aspiration. *Trial Registration*: This trial was registered at www.pactr.org and the identification number for the registry is PACTR202006612296119.

**Results:**

During the 12-month study period, this study included 30 eyes (24 patients) out of 1356 phaco surgeries with postphacoemulsification nonplanar Descemet's membrane detachment. Six patients had DMD in both eyes, eight patients had DMD in the right eye, and ten patients had DMD in the left eye. All patients have successful surgeries without complications. The calculated incidence rate for visually significant DMD was 2.2% per year. The mean ± SD time interval from cataract surgery to the primary intervention was 4.2 ± 1.1 days. Descemet's membrane was attached in 56.25% of patients in group A (9 out of 16 eyes) and 92.6% of patients in group B (13 out of 14 eyes) with a minimum of one-month follow-up.

**Conclusion:**

Air descemetopexy combined with the internal fluid aspiration demonstrated to be more efficient than air descemetopexy only to treat Descemet's membrane detachment following phacoemulsification. It should be tried before planning other major surgeries in patients with severe Descemet's membrane detachment.

## 1. Introduction

Descemet's membrane detachment (DMD) is a severe postphacoemulsification complication that causes permanent corneal damage and decompensation. Even though developments in phacoemulsification innovation made it likely to do cataract surgery with microincision and get preferable postoperative results, a relatively high incidence of DMD has been recorded [1]. The incidence of clinically significant postphacoemulsification Descemet's membrane detachment (DMD) varies from 0.044 to 0.5% [2, 3].

The potential causal factors include corneal dystrophy, shallow anterior chambers, complicated surgeries, dense nuclear cataracts, and accidental injection of viscoelastic substance or saline between Descemet's membrane and stroma [4–7]. Although rare cases of spontaneous reattachment of Descemet's membrane have been recorded [7], surgical interference to enhance reattachment is the standard method for most patients. Early intervention is important to avoid the wrinkling fibrosis and shrinkage of Descemet's membrane and to achieve good visual rehabilitation [8].

There is no gold standard for the treatment options of DMDs. Alternatives include observation, topical steroids, hyperosmotic agents, intracameral air or expandable gas injection, viscoelastic injection, transcorneal suturing, endothelial keratoplasty, and penetrating keratoplasty [9].

This study presents an alternative technique to treat postphacoemulsification Descemet's membrane detachment. It compares air bubble tamponade alone with air bubble tamponade with the internal fluid aspiration after clear corneal incision phacoemulsification.

## 2. Methods

This study is a prospective, intervention, comparative randomized clinical trial, conducted at a private eye centre, Ismailia, Egypt, from March 2019 to March 2020. It was registered at http://www.pactr.org and the identification number for the registry is PACTR202006612296119.

In this study, we followed the classification proposed by Mackool and Holtz [10], which categorised DMD into planar (separation <1 mm) and nonplanar (separation >1 mm) from the corneal stroma. They divided whether involvement is limited to the periphery of the cornea or involves the periphery and the central area of the cornea.

It contained 30 eyes of 24 patients who had postphacoemulsification nonplanar Descemet's membrane detachment involving the periphery and the central area of the cornea (>50% of the cornea) with corneal oedema.

Patients with nonplanar DMD affecting central cornea were divided randomly into two groups: group A treated by air bubble tamponade only and group B treated by air bubble tamponade augmented by the internal fluid aspiration.

Patients' eyes were randomly allocated to group A or group B using the simple randomization procedure. Allocation sequence was generated using the RAND () function in Microsoft Excel software (version 2016).

### 2.1. Inclusion Criteria

All the patients had coaxial phaco surgeries, through a 3 mm superior clear corneal incision with a single-use metal keratome and used Viscomed (hydroxypropyl methylcellulose 2.4%; Albomed GmbH, Germany) as an ophthalmic viscosurgical device. All patients had no previous intraocular surgery.

On the first postoperative day, patients with DMD were diagnosed on clinical grounds through slit-lamp examination. Anterior segment optical coherence tomography (Optovue RTVue, California, USA) was taken in all patients to confirm the diagnosis and determine the type and the extent of the detachment. Patients with nonplanar DMD involving >50% of the cornea with central corneal involvement were included in the study.

### 2.2. Surgical Intervention

The interferences were completed under topical anaesthesia in the operation room to drain the predescemetic fluid joined with intracameral air bubble tamponade. The periocular area was sterilised with 10% povidone-iodine, and an anterior chamber paracentesis was performed in an area of attached DM. A 30-gauge hydrodissection cannula on a 2 cc syringe containing air was used to go below the detached DM, and the anterior chamber was filled with air. The paracentesis was fixed with a cotton wipe for about 1 minute to keep the air inside the anterior chamber and prevent it from getting away out. In group B patients, extra paracentesis was made with a 30-gauge needle at the peripheral cornea to aspirate the internal fluid between the cornea and detached Descemet's membrane using the result of anterior segment optical coherence tomography (AS-OCT) as a guide. The needle stopped shortly after it penetrated the corneal stroma. The air was injected again into the anterior chamber through the initial paracentesis to push the predescemetic fluid out, along with aspiration with the syringe.

**Figure 1:**
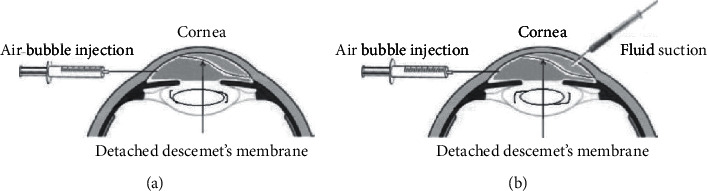
Schematic illustration of the surgical technique in both groups. (a) Group A. (b) Group B.

The anterior chamber was totally loaded up with air. Patients were kept in a supine position for 15 minutes, after which partial relief of air was done so that the anterior chamber was air-filled for almost two-thirds of its volume (Figure 1).

All processes followed the Declaration of Helsinki and adhered to CONSORT guidelines. It was approved by the Research Ethics Committee of the Faculty of Medicine, Suez Canal University. Written informed consent was obtained from the patients. The outcome was assessed as (1) anatomical success: reattachment of Descemet's membrane and (2) functional success: the resolution of corneal oedema.

The same surgeon (AAG) treated and followed all the patients in the two groups.

### 2.3. Postintervention Evaluation

The patients were advised to be in the supine position for the first day after the intervention. A combination of 0.3% tobramycin and 0.1% dexamethasone eye drops three times/day for the first week, decreased once/week, and antiglaucoma (brimonidine 0.1%) eye drops three times/day was advised. Topical tropicamide 1% eye drops were given twice daily till complete resolution of the air bubble.

On the first postintervention day, the slit-lamp examination, IOP was noted. On follow-up at the first week and in the fourth week, the parameters noted were slit-lamp examination, IOP, BCVA, and AS-OCT to examine DM reattachment and complications of the intervention. If, after the primary intervention, there was significant persistent DMD, reintervention was arranged. The time interval between the primary intervention and reinterventions was also recorded.

### 2.4. Statistical Analysis

All data handling and analysis were accomplished by Statistical Package for the Social Sciences (SPSS version 25.0; IBM Corporation, Armonk, NY, USA). Baseline characteristics of the study were presented as percentages and frequencies, or mean and standard deviations. The differences between frequencies in the two groups were compared through the chi-square test or Fisher's exact test (if >20% of expected values were less than 5). The differences in quantitative measurements between the two groups were tested for statistical significance using the Mann–Whitney test. A Wilcoxon signed-rank test was used to compare the preintervention and postintervention measurements. A *p* value < 0.05 was considered statistically significant. The graphs were performed with GraphPad Prism (version 5.00 for Windows, GraphPad Software, La Jolla, California USA). Shapiro–Wilk's test was used to test for data normality.

## 3. Results

**Figure 2:**
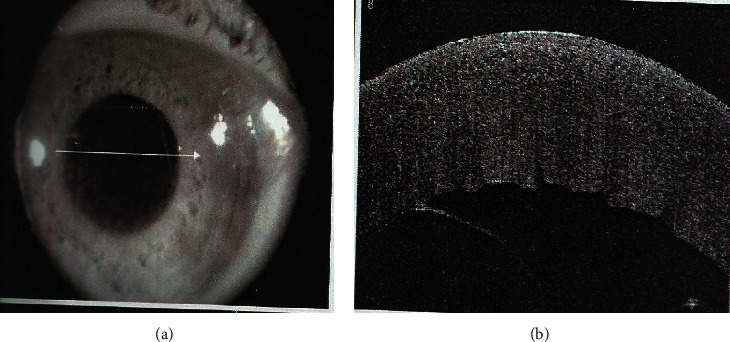
Preintervention nonplanar Descemet's membrane detachment affecting the central cornea with severe corneal oedema.

This study included 30 eyes (24 patients) out of 1356 phacoemulsification surgeries with postphacoemulsification nonplanar Descemet's membrane detachment (Figure 2). All cases were coaxial phaco surgeries, by 2.4 mm superior clear corneal incision fashioned with a disposable metal keratome and Optiflex (hydroxypropyl methylcellulose 2%; Moss Vision Inc. Ltd., UK) as the viscoelastic device. Six patients had DMD in both eyes, eight patients had DMD in the right eye, and ten patients had DMD in the left eye. The mean ± SD of the age of the patients was 62 ± 2.1 years and 61 ± 3.2 years in groups A and B, respectively. The male-to-female ratio was 38.5 : 61.5 in group A and 45.5 : 54.5 in group B. All the patients have successful surgeries without complications with a minimum of 1-month follow-up. The calculated incidence rate per year (study time) for visually significant DMD was 2.2% (this rate does not include mild cases treated medically only). The patients were divided into two groups: group A included 16 eyes and group B included 14 eyes (Figure 1). The demographic data of both groups A and B are presented in Table 1.

### 3.1. DMD Site

DMD originated from main corneal incision was made in 23 eyes (76.7%): 11 eyes (36.7%) in group A and 12 eyes (40%) in group B. Main and side incisions were made in 5 eyes (16.6%): 3 eyes (10%) in group A and 2 eyes (6.6%) in group B. Side incisions were made in 2 eyes (6.7%): 1 eye (3.35%) in group A and 1 eye (3.35%) in group B. Central cornea (>50% of the cornea) was involved in all cases.

### 3.2. Time of Resolution

The mean ± SD of the time interval from phacoemulsification surgery to the primary intervention was 4.2 ± 1.1 days in group A and 4.3 ± 0.9 days in group B. After air descemetopexy, the DM was attached in 9 eyes (56.25%) in group A and 13 eyes (92.6%) in group B with a minimum of 1-month follow-up. There was a significant difference in the success rates between the two groups of patients (*p*=0.039). The time of resolution was shorter in group B as compared to group A with a significant difference (*p*=0.044^*∗*^) (Table 2). Six eyes of group A and one eye of group B needed secondary reinterventions. The mean ± SD of the time interval for the reintervention was 9 ± 2.1 days. Patients who required reintervention were followed up for one month with successful reattachment of DM in 100% of all patients.

### 3.3. Visual Recovery

**Figure 3:**
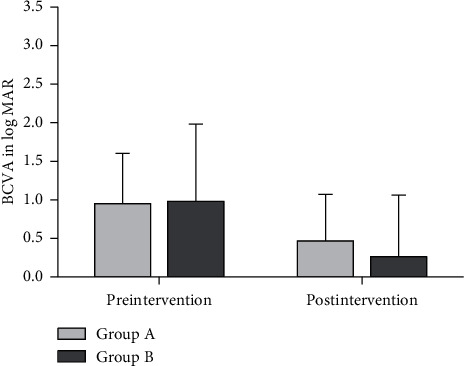
Pre- and postintervention mean of the log MAR of BCVA at one month in both groups.

The mean ± SD of the log MAR of the BCVA in both groups was improved from 0.95 ± 0.65 and 0.98 ± 1.0 to 0.47 ± 2.1 and 0.26 ± 1.5 in groups A and B, respectively, with significant *p* values (0.042^*∗*^ and 0.048^*∗*^) (Table 3 and Figure 3). Out of the 13 patients with attached DM in group A, 10 patients had improvement in BCVA 3 Snellen lines, and out of 11 patients in group B, 10 patients had improvement in BCVA 4 Snellen lines.

### 3.4. Corneal Thickness

**Figure 4:**
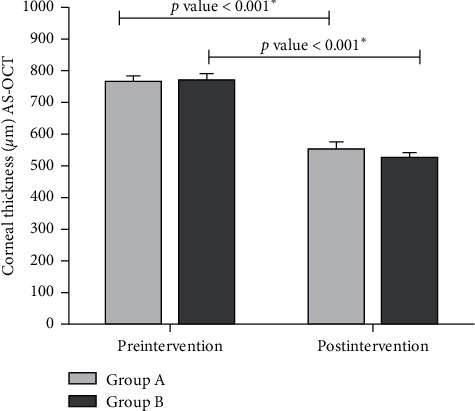
Pre- and postintervention mean of the central corneal thickness (CCT) of both groups at one month after the intervention.

**Figure 5:**
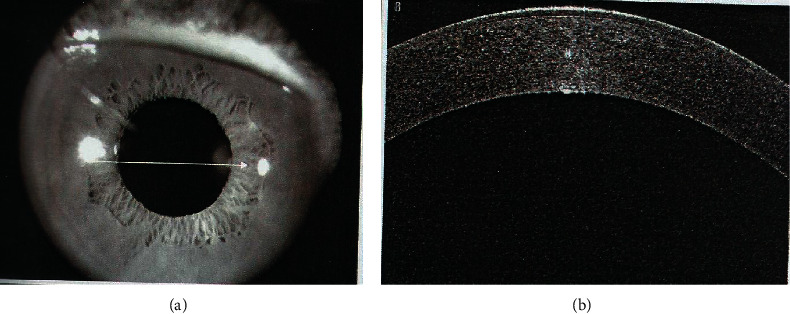
Postintervention with complete Descemet's membrane reattachment and resolution of corneal oedema.

The mean ± SD of the corneal thickness was 766 ± 18 *μ*m and 771 ± 20 *μ*m improved to 554 ± 22 *μ*m and 527 ± 15 *μ*m in groups A and B, respectively (*p*=0.001^*∗*^) at one month after the intervention (Table 3 and Figures 4 and 5).

### 3.5. Complications

All the patients had within normal IOP after the primary and the secondary reinterventions. No corneas decompensated during the follow-up of the study.

## 4. Discussion

Descemet's membrane detachment (DMD) is defined as separating Descemet's membrane from the posterior corneal stroma. Mild DMD is visually insignificant, commonly overlooked (or if diagnosed), which is managed with topical hyperosmotic agents. More likely, they get spontaneously reattached after some time [11].

The main corneal incision possibly experiences forceful fluid currents through a snug-fitting between the phaco machine's probe and the clear corneal incision, therefore a higher risk of accidental DM stripping, mainly at this site [12].

Weng et al. [13] reported in this case study that drainage of predescemetic fluid joined with intracameral air bubble tamponade was another surgical alternative for managing a severe case of DMD. This study is the first yet reported series of postphacoemulsification nonplanar DMD, with almost 92.6% of the patients attaining successful reattachment of DM with air bubble tamponade augmented with internal fluid aspiration versus 56.25% in patients with only air tamponade. Also, the time of resolution was faster in group B versus group A with a significant difference (*p*=0.044^*∗*^). Nonplanar DMD, to some extent, resembles a serous retinal detachment in appearance. When the surgeon is doing stromal hydration after a successful phacoemulsification, it is normal to see this. Holding the cannula close to the posterior stroma may cause the fluid wave to spread between the stroma and Descemet's membrane causing DMD, which is basically fluid stuck under Descemet's membrane as a slightly convex (fluid pocket) into the anterior chamber (Figure 2). An exit must be created for the fluid to evacuate completely and the detachment to fully decompress with a tamponade agent to allow reattachment. The injected air in the anterior chamber raises the detached Descemet's membrane up to adhere to the stroma of the cornea. At the same time, the drainage of predescemetic fluid externally by syringe aspiration (analogous to internal tamponade and subretinal fluid drainage in retinal detachment) helps Descemet's membrane stick to the collagen of the cornea and prevents the detachment from more expansion. It is simple to understand that air bubble alone will not completely work. This allows the membrane to adhere faster and corneal oedema to improve earlier and visual acuity improvement with a minimally invasive surgical manipulation to the cornea. This is considered an important finding, suggesting that air tamponade with internal fluid aspiration is worth trying, especially in severe cases of DMD. This helps to prevent major surgeries such as penetrating keratoplasty.

The calculated incidence rate for visually significant DMD in this study was 2.2% per year (30 out of 1356 phacoemulsification surgeries). This is a relatively high incidence rate compared to other studies [2, 5, 8, 14, 15]. This could be explained by the excessive wound hydration, hardness of the cataract, hot climate, or late cataract surgeries for economic reasons. Genetic factors (preoperative corneal dystrophies) may play a role in this high rate. Relevant to that, Anderson [16] reported a 5% incidence rate of DMD, and Jaramillo et al. [17] reported a 7.4% incidence rate of DMD. Some authors reported up to 43% incidence rate of DMD [18].

Air is usually preferred as a tamponading agent for many causes, including its lower cost, shorter absorption time, and low risk of endothelial toxicity or pupillary block than with other long-standing gases including 14% perfluoropropane and 20% sulphur hexafluoride [19, 20]. Tamponading with viscoelastic agents has been reported in some previous studies [21, 22] as a treatment option for DMD, but this method has some limitations because of the high risk of elevating the intraocular pressure and the need for continuous follow-up and monitoring [3].

One limitation of this study was underestimation of the true incidence of DMD since only moderate and severe nonplanar DMD were included, while mild cases were not, which are visually insignificant (usually missed or are managed with topical hyperosmotic agents). Second, the relationship between the grade of the cataract and the incidence of DMD was not assessed. Last, whether the success of surgeries is affected to some extent by the state of the endothelium which needs further study on the preoperative assessment by the specular microscopy.

In conclusion, air descemetopexy combined with internal fluid aspiration demonstrated to be more efficient than air descemetopexy only to treat Descemet's membrane detachment following phacoemulsification. It should be tried before planning other major surgeries in patients with severe Descemet's membrane detachment.

## Figures and Tables

**Table 1:** Preoperative demographic data for both groups.

Demographic data	Group A (*n* = 16 eyes, 13 ptns)	Group B (*n* = 14 eyes, 11 ptns)	*p* value
*Eye*			
Rt (*n*)	6	8	0.290
Lt (*n*)	10	6	

*Bilaterality*			
Bilateral (*n*)	4	2	0.640
Unilateral (*n*)	8	10	

*Sex*			
Male, *n* (%)	5 (38.5%)	5 (45.5%)	0.735
Female, *n* (%)	8 (61.5%)	6 (54.5%)	

*Age* (*years*)			
Mean ± SD	62 ± 2.1	61 ± 3.2	0.426

*BCVA* (*log MAR*)			
Mean ± SD	0.95 ± 0.65	0.98 ± 1.0	0.982

*Notes*. Group A: patients with nonplanar DMD affecting the central cornea treated by air bubble tamponade only; group B: patients with nonplanar DMD affecting the central cornea treated by air bubble tamponade with internal fluid aspiration. *n*: number; SD: standard deviation; ptns: patients.

**Table 2:** Postoperative success rates of both groups.

Parameter	Group A, *n* = 16	Group B, *n* = 14	*p* value
Time of intervention: mean ± SD (days)	4.1 ± 1.1	4.3 ± 0.9	0.623
Time of resolution: mean ± SD (days)	1.9 ± 1.2	1.1 ± 0.8	0.044^*∗*^
Reattachment: *n* (%)	9 (56.25%)	13 (92.6%)	0.039^*∗*^

*Notes*. Group A: patients with nonplanar DMD affecting the central cornea treated by air bubble tamponade only; group B: patients with nonplanar DMD affecting the central cornea treated by air bubble tamponade with internal fluid aspiration. SD: standard deviation; *n:* number; BCVA: best-corrected visual acuity. ^*∗*^Statistically significant (*p* value <0.05).

**Table 3:** Pre- and postintervention log MAR of BCVA and corneal thickness in both groups at one month after the intervention.

Measurement	Time	Group A	Group B	*p*1 value
BCVA in log MAR	Preintervention: mean ± SD	0.95 ± 0.65	0.98 ± 1.0	0.965
	Postintervention: mean ± SD	0.47 ± 0.6	0.26 ± 0.8	0.520
	*p*2 value	0.042^*∗*^	0.048^*∗*^	

Corneal thickness (*μ*m) AS-OCT	Preintervention: mean ± SD	766 ± 18	771 ± 20	0.512
	Postintervention: mean ± SD	554 ± 22	527 ± 15	<0.001^*∗*^
	*p*3 value	<0.001^*∗*^	<0.001^*∗*^	

*Notes*. Group A: patients with nonplanar DMD affecting the central cornea treated by air bubble tamponade only; group B: patients with nonplanar DMD affecting the central cornea treated by air bubble tamponade with internal fluid aspiration. SD: standard deviation; BCVA: best-corrected visual acuity; AS-OCT: anterior segment optical coherence tomography. ^*∗*^Statistically significant.

## Data Availability

The datasets used and/or analysed during the current study are available from the corresponding author on reasonable request.

## Supplementary Materials

Supplementary Materials CONSORT 2010 checklist of information to include when reporting a randomised trial.
